# Evaluation of Ultrasonographic Optic Nerve Sheath Diameter as a Point-of-Care Tool for Assessing Intracranial Pressure in Pediatric Patients With Hydrocephalus Undergoing Neurosurgical Intervention

**DOI:** 10.7759/cureus.91261

**Published:** 2025-08-29

**Authors:** Malika Dhawal, Hemlata Hemlata, Ahshan Siddiqui, Monica Kohli

**Affiliations:** 1 Anaesthesia and Critical Care, King George’s Medical University, Lucknow, IND

**Keywords:** hydrocephalus, intracranial pressure, optic nerve sheath diameter, pediatric, ultrasound

## Abstract

Background

The optic nerve sheath diameter (ONSD) on ocular ultrasonography has recently emerged as a reliable and useful indirect tool for measuring raised intracranial pressure (ICP). However, findings of previous studies have been inconsistent or inconclusive, and not many studies have evaluated its utility in the pediatric population undergoing neurosurgical interventions. This observational study aimed to evaluate the efficacy of ultrasonographic ONSD as a point-of-care tool for predicting real-time ICP in children with hydrocephalus undergoing shunt surgery.

Methodology

This study included 52 children aged 2 months to 10 years with hydrocephalus undergoing ventriculoperitoneal shunt under general anesthesia. Ultrasonographic ONSD for each eye and invasive ICP were measured before surgery (after induction) and after surgery (before extubation). Statistical analyses were performed using SPSS version 21 (IBM Corp., Armonk, NY, USA).

Results

Preoperatively, the mean ONSD in the right and left eyes was 5.75 ± 1.6 mm and 5.68 ± 1.35 mm, respectively, which reduced significantly to 4.3 ± 0.89 mm and 4.48 ± 0.79 mm, respectively, postoperatively. The correlation between ONSD and raised ICP was significantly positive (p < 0.001, r = 0.879). Analysis of the receiver operating characteristic curve revealed that a cutoff ONSD value of 5.5 mm predicted an ICP ≥20 mmHg with a specificity of 75%, sensitivity of 100%, and a positive predictive value of 95%. These results confirm that elevated ONSD is suggestive of raised ICP.

Conclusions

Ultrasonographic ONSD is a useful non-invasive point-of-care tool for the assessment and monitoring of children with hydrocephalus suspected of having raised ICP.

## Introduction

The gold standard for the diagnosis of intracranial hypertension is direct monitoring of intracranial pressure (ICP) using an intracranial catheter [1]. However, being invasive, this procedure carries the risk of infection and other serious complications. Recently, ultrasonographic measurement of optic nerve sheath diameter (ONSD) has emerged as a reliable and indirect tool for detecting acute changes in ICP [2]. The mechanism of enlargement of the ONSD with an increase in ICP is well-known. The eyeball is directly connected to the cranium through the optic nerve and the surrounding optic nerve sheath (ONS), which are cylindrical structures running posterior-centrally and slightly upward toward the optic chiasma. The ONS surrounds the optic nerve and is filled with cerebrospinal fluid (CSF), communicating directly with the subarachnoid space. Raised ICP causes enlargement of the optic nerve that is reflected in ultrasonographic measurement of ONSD [2,3].

The invasive positioning of ventricular or intraparenchymal devices makes them the gold standard for accurate ICP monitoring. Among these, the external ventricular drain (EVD) coupled to an external fluid-filled transducer remains the best choice both for its measurement accuracy and for allowing therapeutic CSF drainage at the same time [4]. The use of point-of-care ultrasound (POCUS) for diagnostic assessment has recently become widespread in pediatric emergency and critical care services, representing a valuable extension of the physical examination [5].

The intraorbital portion of the optic nerve is ontogenetically a part of the central nervous system and extends from the ocular bulb to the optic canal. It is surrounded by a membrane continuous with the dura mater of the brain, called ONS, and contains CSF. As the ONS is distensible, acute variations of CSF pressure lead to changes in ONSD within minutes [6-8].

The ideal cut-off value for an elevated ONSD, suggesting raised ICP, remained unknown, as only a limited number of healthy people were involved in most research on ONSD calculation. In addition, while previous studies have examined ONSD-related demographic and physiological factors, the findings have been inconsistent or inconclusive. Furthermore, some studies focus only on adults or mixed populations, and many of these only evaluate the change in ultrasonographic ONSD without correlating it with invasive ICP measurement. Though age-specific cut-offs for pediatric patients have been proposed in some previous studies, they were done in different sets of populations and not in children with hydrocephalus.

Hence, we aimed to conduct this study to evaluate the efficacy of ultrasonographic measurements of ONSD in predicting real-time ICP dynamically and sensitively in children with hydrocephalus undergoing shunt surgery. The primary objective was to assess the change in ONSD after surgical intervention, and the secondary objectives were to correlate the ultrasonographic ONSD with invasive ICP measurement, monitor hemodynamic parameters, and note complications, if any.

This article was previously presented as a paper at the Annual UPISACON 2022 on September 23, 2022, in Noida, India, and as a poster at the ANZCA Annual Scientific Meeting in May 2024 in Brisbane, Australia.

## Materials and methods

This prospective, observational study was conducted in the Department of Anesthesiology, in collaboration with the Department of Neurosurgery at King George’s Medical University, Lucknow, after obtaining approval from the Institutional Ethical Committee (approval number: ECR/262/Inst/UP/2013/RR-19) and written informed consent from parents and guardians. The study included 52 pediatric patients with hydrocephalus aged between 2 months to 10 years, belonging to American Society of Anesthesiologists (ASA) grades I and II, undergoing ventriculoperitoneal shunt surgery lasting less than four hours. Exclusion criteria included patients undergoing emergency neurosurgeries, those with eye or orbit disease, such as glaucoma and lens opacity, and unwilling patients/guardians.

A preoperative preliminary assessment was performed on all patients, and screening was done as per the inclusion and exclusion criteria. The ultrasonographic ONSD measurement was taken before invasive ICP measurement in children after the induction of general anesthesia. The ultrasonographic ONSD measurements were performed in the supine position by two experienced anesthesiologists who were unaware of each other’s results. The measurement was done using a 14-5 MHz probe (Sonoscope S-50) and B-mode. The probe was placed on the closed upper eyelid, and the angle was adjusted to display the optic nerve entry into the eyeball. The ONSD was measured at a depth of 3 mm behind the eye globe, and the mean of the measurements by the two anesthesiologists was taken for each eye. During surgery, invasive ICP was measured by the surgeon using a 16-gauge intravenous cannula. The catheter end of the cannula was inserted into the ventricular end of the medium-pressure ventriculoperitoneal shunt, and the cannula’s distal end was connected to pressure monitoring lines that were transduced to the pressure transducer system, taking great care in preventing any loss of CSF, and ICP was recorded. ONSD was again measured at the end of surgery before extubation. For each patient, age, sex, weight, Glasgow Coma Scale (GCS) status, ASA grade, and duration of procedure were noted.

Sample size was calculated based on variation in ONSD among the cases with and without raised ICP using the following formula: n = 2 (z_α_ + z_β_ )^2 ^(s_1_^2 ^+ s_2_^2^ )/d^2^, where s_1_ = 0.5, the SD of ONSD among the cases without raised ICP; s_2_ = 0.6, the SD of ONSD among the cases with raised ICP in a study by Newman et al. [9]; d = min (s_1_, s_2_), the difference considered to be clinically significant; type I error α = 5% corresponding to a 95% confidence level; and type II error β = 20% for detecting results with 80% study power. Minimal clinically important difference was taken as the minimum of two SDs of ONSD among cases with and without raised ICP.

For statistical analysis, we used SPSS version 21 software (IBM Corp., Armonk, NY, USA). Mean (standard deviation) or range was used to evaluate continuous variables when required. The dichotomous variables were presented in number/frequency, and the chi-square or Fisher’s exact test was used to analyze these variables. For comparing the means of two groups, Student’s t-test, Mann-Whitney U test, and Spearman’s correlation with a 95% confidence interval (CI) were used. A p-value <0.05 was considered significant.

## Results

**Figure 1:**
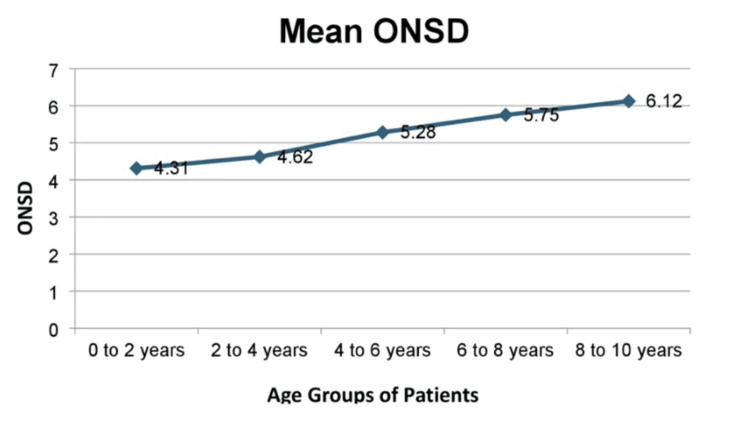
Correlation between age group and mean ONSD. ONSD values are in mm. ONSD: optic nerve sheath diameter

This study was conducted among 52 pediatric patients with hydrocephalus undergoing ventriculoperitoneal shunt, of whom 33 (63.46%) were male and 19 (36.53%) were female. Among the 52 patients, 18 were under two years of age with an ONSD of 4.31 ± 0.89 mm, 16 patients were aged 2-4 years with an ONSD of 4.62 ± 0.92 mm, 11 patients were aged 4-6 years with an ONSD of 5.28 ± 1.03 mm, and five patients were aged 6-8 years with an ONSD of 5.75 ± 1.08 mm, and two patients were aged 8-10 years with an ONSD of 6.12 ± 1.12 mm. The ultrasonographic ONSD and invasive ICP were measured at predefined time points, and the recorded data were analyzed. The evaluation of the mean ONSD of different age groups showed a positive and significant correlation with age (increasing trend of ONSD with respect to increasing age group) (Table 1, Figure 1).

**Table 1:** Correlation between age group and mean ONSD. P-value was calculated using the chi-square test. ONSD: optic nerve sheath diameter

Age	Number of subjects	Mean ONSD (mm)	r value	P-value
0–2 years	18 (34.61%)	4.31 ± 0.89	0.412	0.032
2–4 years	16 (30.76%)	4.62 ± 0.92		
4–6 years	11 (21.15%)	5.28 ± 1.03		
6–8 years	5 (9.61%)	5.75 ± 1.08		
8–10 years	2 (3.84%)	6.12 ± 1.12		
Total	52 (100%)	5.29 ± 1.17	-	-
Mean age	4.21 ± 3.75		-	-

Additionally, the mean ONSD value in age groups less than two years and more than two years was compared, and a significantly positive correlation was noted (Table 2).

**Table 2:** Correlation between age group below and above two years and mean ONSD. P-value was calculated by Student’s t-test. ONSD: optic nerve sheath diameter

Age	Number of subjects	Mean ONSD (mm)	r value	P-value
Below 2 years	18 (34.61%)	4.31 ± 0.89	0.212	0.049
Above 2 years	34 (65.38%)	5.44 ± 0.21		
Total	52 (100%)	5.29 ± 1.17	-	-
Mean age	4.21 ± 3.75		-	-

Preoperatively, the mean ONSD was 5.75 ± 1.6 mm on the right side and 5.68 ± 1.35 mm on the left side. Postoperatively, the mean ONSD was 4.3 ± 0.89 mm on the right side and 4.48 ± 0.79 mm on the left side. There was a statistically significant decrease in mean ONSD bilaterally after surgical intervention (Table 3).

**Table 3:** Correlation between preoperative and postoperative ONSD. P-value was calculated by Student’s t-test. ONSD: optic nerve sheath diameter

	Preoperative ONSD (mm)	Postoperative ONSD (mm)	r value	P-value
Right	5.75 ± 1.6	4.3 ± 0.89	0.421	<0.001
Left	5.68 ± 1.35	4.48 ± 0.79	0.455	<0.001

There was a statistically significant decrease in invasive ICP postoperatively from the preoperative value in all the patients (Table 4).

**Table 4:** Comparison of preoperative and postoperative ICP. P-value was calculated by Student’s t-test. ICP: intracranial pressure

Intracranial pressure	Mean ± SD (mmHg)	P-value
Preoperative	20.31 ± 7.32	<0.001
Postoperative	18.32 ± 5.63	

**Figure 2:**
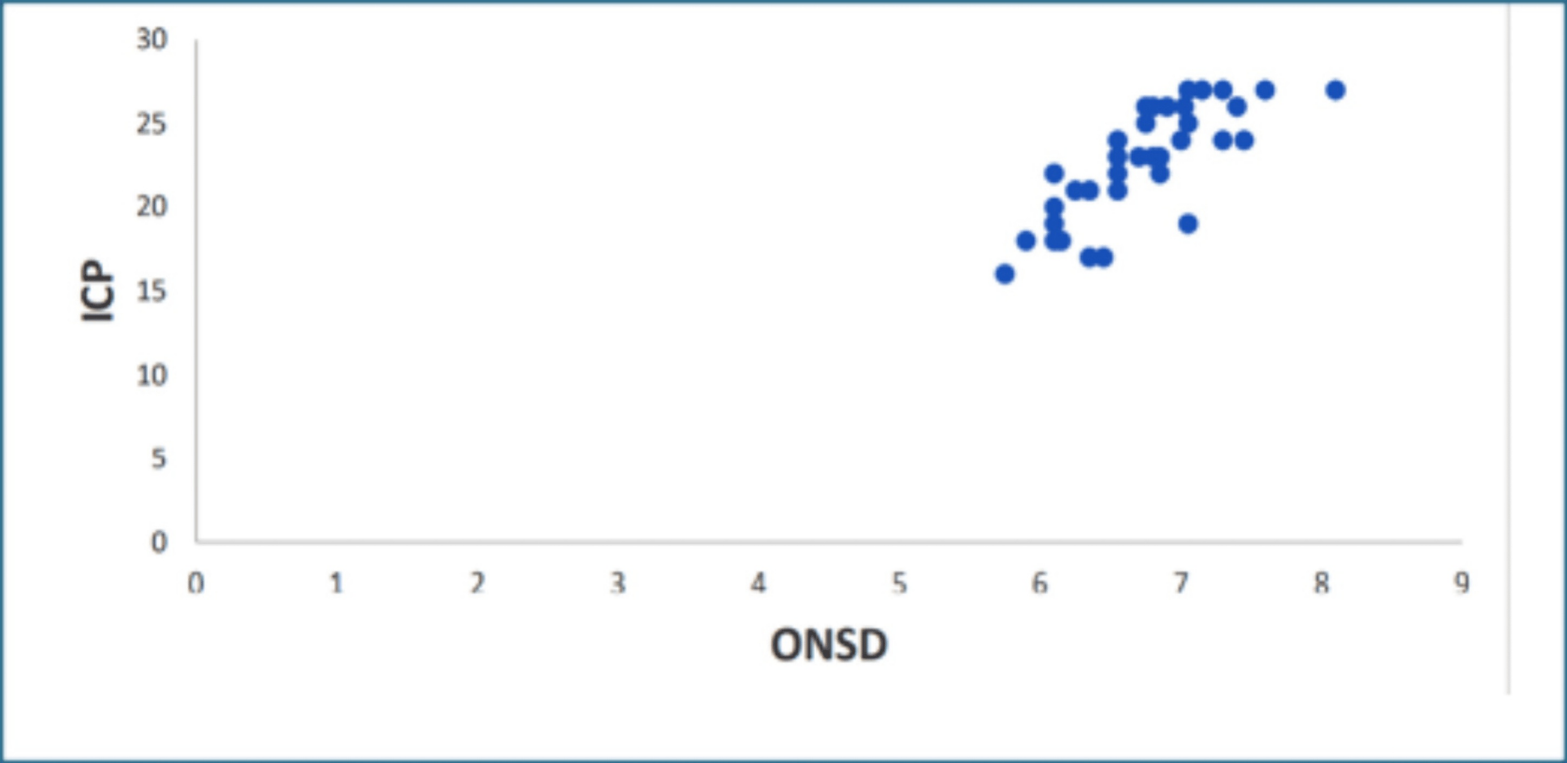
Correlation between ONSD and ICP. Pearson’s correlation coefficient. ICP is the dependent variable, and ONSD is the independent variable. ONSD: optic nerve sheath diameter; ICP: intracranial pressure

Regression analysis for ICP and ONSD showed a significant linear correlation between ICP and ONSD (Table 5, Figure 2).

**Table 5:** Correlation between ICP and ONSD. ONSD: optic nerve sheath diameter; ICP: intracranial pressure; SD: standard deviation

Regression analysis for ICP and ONSD	
R square	0.879
Intercept	-11.87
X variable	5.094
SD	0.71
P-value	<0.001

**Figure 3:**
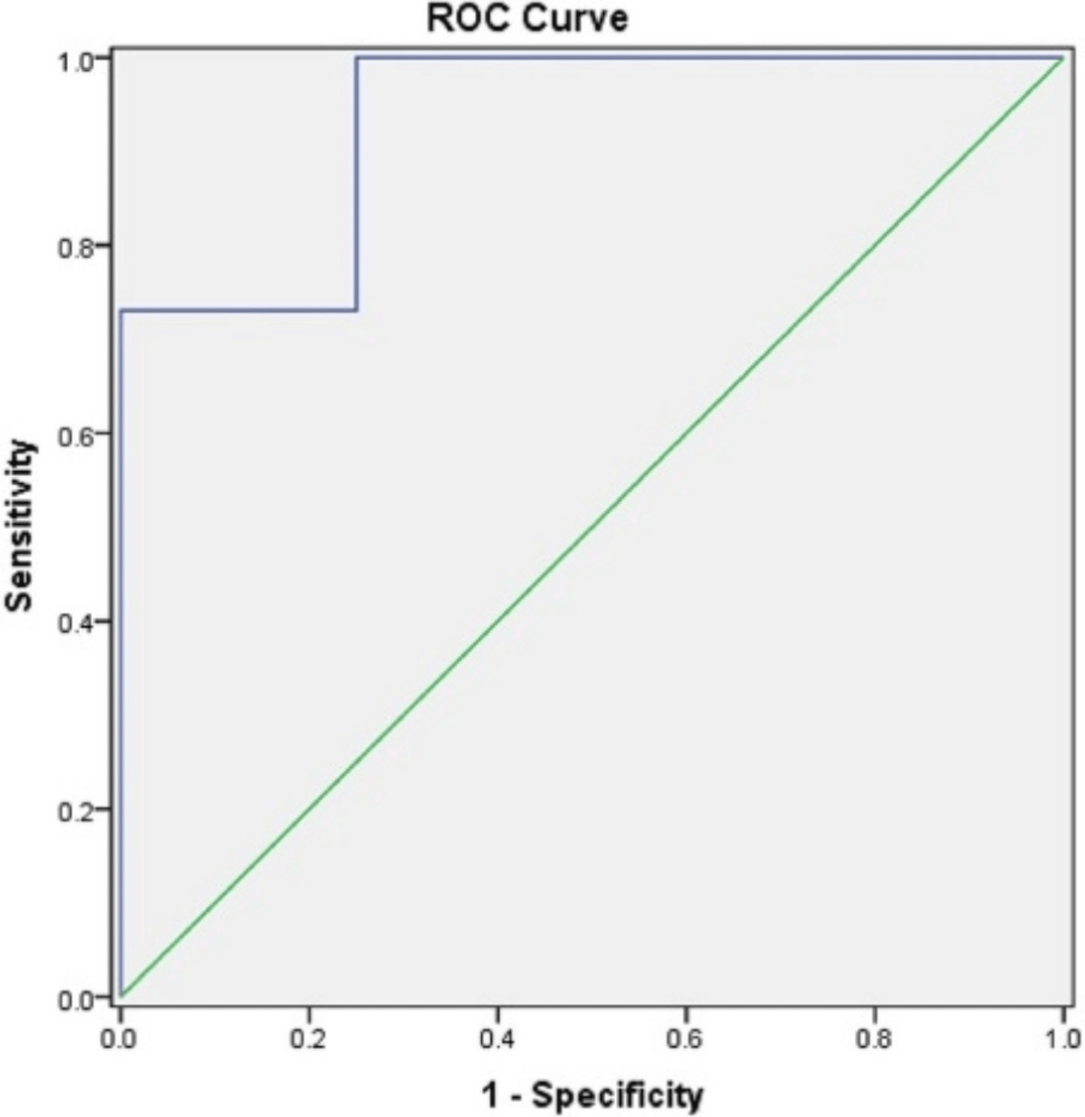
ROC curve for ICP. ROC: receiver operating characteristic; ICP: intracranial pressure

Analysis of the results of the receiver operating characteristic (ROC) curve revealed that a cutoff ONSD value of 5.5 mm in children aged 2-10 years predicted an ICP ≥20 mmHg with a specificity of 75%, sensitivity of 100%, and a positive predictive value of 95% (Figure 3, Table 6).

**Table 6:** Results of receiver operating characteristic analysis for ICP. AUC: area under the curve; CI: confidence interval; PPV: positive predictive value; ICP: intracranial pressure

Estimator	Value
AUC (95% CI)	0.934
Sensitivity	100.0%
Specificity	75.2%
PPV	95.0%
P-value	0.005

Comparison of various parameters between the groups of patients with preoperative ICP <20 mmHg and those with ICP >20 mmHg revealed that there were no significant differences in mean systolic blood pressure, diastolic blood pressure, mean arterial pressure, respiratory rate, SpO_2_, or EtCO_2_ between the two groups.

## Discussion

In recent years, there has been much interest in identifying and developing reliable, non-invasive, and bedside methods of detecting elevated ICP. The use of ultrasonographic ONSD measurement for the indirect assessment of ICP is already well known [10]. POCUS is preferred as a new method for the detection of increased ICP and regular clinical follow-up as it is easy, fast, cheap, repeatable, and non-invasive [11,12]. As a rapid and non-invasive method, it has become a new tool for pediatricians as well in emergency and intensive care departments for monitoring elevated ICP [13]. As the ONS is continuous with the meninges and subarachnoid space, the ONSD increases in patients with increased ICP [14]. Early detection of raised ICP is considered a better strategy to prevent secondary brain insults.

In our study, 52 patients were enrolled between the ages of 2 months and 10 years, with 33 males and 19 females. We found congenital hydrocephalus to be more common in males than in females in our pediatric population. Among the 52 patients, 18 were aged under two years with an ONSD of 4.31 ± 0.89 mm, 16 were aged 2-4 years with an ONSD of 4.62 ± 0.92 mm, 11 were aged 4-6 years with an ONSD of 5.28 ± 1.03 mm, five were aged 6-8 years with an ONSD of 5.75 ± 1.08 mm, and two were aged 8-10 years with an ONSD of 6.12 ± 1.12 mm.

In our study, preoperative and postoperative ONSD were recorded for both eyes using ultrasound. We compared the preoperative mean ONSD (5.75 ± 1.6 mm on the right side and 5.68 ± 1.35 mm on the left side) with postoperative mean ONSD (4.3 ± 0.89 mm on the right side and 4.48 ± 0.79 mm on the left side), and found a statistically significant reduction. We also correlated the ultrasonographic ONSD with invasive ICP measurement and found a positive significant linear correlation between the two.

In a study by Rehman Siddiqui et al. [15] based on the clinical signs of raised ICP, such as GCS <9-13, anisocoric pupil, motor posturing, patients with the Cushing’s triad, and with neurological symptoms, a surrogate marker of raised ICP in 48 patients, they identified the threshold of ultrasonographic ONSD measurement in infants as >4.0 mm, and in children 1-10 years as >4.71 mm, with a sensitivity of 100% and a specificity of 60-66.7%. Overall, 85% of patients with GCS ≤12 showed raised ICP with ultrasonographic ONSD measurement, which was nearly similar to our study, in which the mean ONSD in the <2-year age group was 4.31 ± 0.89 mm and the mean ONSD in the >2-year age group was 5.44 ± 0.21mm, with significant correlation between the age groups and ONSD measurements.

In a study by Ballantyne et al. [16], 102 normal healthy children up to 15 years were included. The range for ONSD using ultrasonography was 2.1-4.3 mm, with a mean ONSD of 3.08 ± 0.36 mm (SD). They analyzed the correlation between increasing age and increasing ONSD. Their results suggested that an ONSD >4 mm in those aged less than one year and 4.5 mm or greater in older children should be considered abnormal. In our study, we found a correlation between the age group and ONSD in patients with hydrocephalus. Patients with raised ICP with age <2 years had an ONSD of 4.31 mm, and those aged >2 years had an ONSD of 5.44 mm.

Ultrasound is a bedside method that is safe, non-invasive, repeatable, and provides information about ICP rapidly is useful in the emergency setting. It helps in the follow-up of patients by quick assessment of the variation in ICP when invasive monitoring devices are contraindicated or not available, especially in resource-limited settings. Thus, ultrasonographic ONSD measurement can be used in combination with clinical signs of raised ICP to detect intracranial hypertension during secondary insult to head injury. Invasive monitoring is normally considered a gold standard reference for comparing other modalities, but differences in the type of invasive devices, site of insertion (intraventricular, intraparenchymal, subdural, epidural), and the location of the tip make it difficult to accurately monitor the exact ICP, especially in localized disease processes.

In another study by Aslan et al. [17], 38 pediatric patients with a mean age of 6.7 ± 5.4 years were included to examine the diagnostic value of ONSD measurements and central retinal artery Doppler indices in the evaluation of pediatric patients with increased ICP. The mean ONSD was 5.9 ± 0.8 (3.6-8.1) mm. The ONSD measurement was found to be the strongest parameter in terms of predicting increased ICP, with an area under the curve (AUC) of 0.767 (95% CI = 0.68-0.85). In our study, we found better correlation, with an AUC of 0.934, with a 95% CI. This may be because our study was conducted exclusively among hydrocephalus patients undergoing ventriculoperitoneal shunt.

Doherty et al. [18] recorded opening intraventricular pressure that predicted the ICP values more accurately and precisely when compared to the aforementioned studies using intraparenchymal probes, EVD, and lumbar CSF pressure. In our study, we used a 16-gauge intravenous cannula; its catheter end was inserted into the distal end of the medium-pressure ventriculoperitoneal shunt, and its distal end was connected to pressure monitoring lines that were connected to the pressure transducer system, and the ICP was recorded. Further, we compared the ultrasonographic ONSD with invasive ICP measurement. Preoperative mean ICP was found to be 20.31 ± 7.32 mmHg, and the postoperative mean ICP was 18.32 ± 5.63 mmHg, with the difference being statistically significant.

ONSD measurement, together with invasive ICP monitoring, is valuable for neurological conditions with clinically suspected variation of ICP and in optic nerve disorders. In our study, we used radiological methods together with clinical methods for measuring ICP.

Most previous studies have been conducted in adult patients. In the future, we can use ultrasonographic ONSD assessment as an alternative investigatory modality in pediatric patients with elevated ICP received at the emergency department.

Early diagnosis and treatment of increased ICP in critically ill children is pivotal to prevent neurological damage. However, the gold standard method of measuring ICP using a catheter placed in the brain parenchyma or ventricle needs expertise in the procedure, besides carrying risks of bleeding and infection; therefore, it is not always possible to implement such a method [19].

In our study, the ultrasonographic ONSD correlated significantly with invasive ICP (r = 0.879, p < 0.001). A cutoff ONSD value of 5.5 mm predicted an ICP ≥20 mmHg, with a specificity of 75%, sensitivity of 100%, and positive predictive value of 95%. In children aged <2 years, the mean ONSD was 4.31 mm, while in children aged >2 years, the mean ONSD was 5.35 mm.

The ultrasonographic measurement of ONSD strongly and accurately predicts raised ICP, and due to its bedside availability, fast application, and high sensitivity, it can be considered for screening patients for raised ICP so that prompt treatment can be provided. Hence, ONSD measurement together with invasive ICP monitoring is valuable for neurological conditions with clinically suspected variation of ICP and in optic nerve disorders.

ONSD measurements using ultrasonography should be obtained preferably by a single experienced anesthesiologist, but in our study, it was not possible for the same person to be available at all times, and hence, measurements were done by more than one trained anesthesiologist, due to which there was potential for interobserver variability that was not evaluated in our study. Another limitation was the relatively small sample size. Therefore, a larger prospective study with children of different age groups should be done in the future to establish the accuracy of ultrasonographic measurement of ONSD as a useful point-of-care tool for the diagnosis of raised ICP in children. Another limitation of ONSD is that it may not reflect raised ICP as accurately in chronic compensated hydrocephalus.

## Conclusions

In our study, we found a significant reduction in ultrasonographic ONSD after surgical intervention in pediatric patients with hydrocephalus, and there was a positive significant linear correlation between ONSD and ICP. We found no significant variation in hemodynamic parameters, and no complications were noted. We conclude that the measurement of ICP through ocular ultrasonography holds potential application for patients visiting the emergency department, who have limitations to undergo physical examinations. Being non-invasive, ultrasonographic ONSD provides a potential tool for the rapid quantification of raised ICP, and together with the clinical methods, it can be used as a point-of-care tool in measurement of ICP, even at the bedside for quick assessment in pediatric patients with neurological deficits.
